# Molecular Interaction Between Vasopressin and Insulin in Regulation of Metabolism: Impact on Cardiovascular and Metabolic Diseases

**DOI:** 10.3390/ijms252413307

**Published:** 2024-12-11

**Authors:** Ewa Szczepanska-Sadowska, Agnieszka Cudnoch-Jędrzejewska, Tymoteusz Żera

**Affiliations:** Department of Experimental and Clinical Physiology, Centre for Preclinical Research, Medical University of Warsaw, 02-097 Warsaw, Poland

**Keywords:** ACTH, brain, cardiovascular system, diabetes mellitus, GLP-1, glucose, insulin, metabolism, obesity, vasopressin

## Abstract

Numerous compounds involved in the regulation of the cardiovascular system are also engaged in the control of metabolism. This review gives a survey of literature showing that arginine vasopressin (AVP), which is an effective cardiovascular peptide, exerts several direct and indirect metabolic effects and may play the role of the link adjusting blood supply to metabolism of tissues. Secretion of AVP and activation of AVP receptors are regulated by changes in blood pressure and body fluid osmolality, hypoxia, hyperglycemia, oxidative stress, inflammation, and several metabolic hormones; moreover, AVP turnover is regulated by insulin. Acting on V1a receptors in the liver, AVP stimulates glycogenolysis, reduces synthesis of glycogen, and promotes fatty acid synthesis and acetyl CoA carboxylase activity. Stimulating V1b receptors in the pancreatic islands, AVP promotes release of insulin and glucagon-like peptide-1 (GLP-1) and potentiates stimulatory effects of glucose and ACTH on secretion of insulin. Simultaneously, insulin increases AVP secretion by neurons of the paraventricular nucleus and the supraoptic nucleus. There is strong evidence that secretion of AVP and its metabolic effectiveness are significantly altered in metabolic and cardiovascular diseases. Both experimental and clinical data indicate that inappropriate interactions of AVP and insulin play an important role in the development of insulin resistance in obesity and diabetes mellitus.

## 1. Introduction

Cardiovascular diseases frequently come with metabolic disorders, and the coincidence of these morbidities worsens the health state and prognosis of the patient [1,2]. Multiple studies show that several factors involved in the regulation of the cardiovascular system are engaged in the regulation of metabolism, but the molecular background of these interactions is not well recognized. Thus far, catecholamines, components of the renin-angiotensin system (RAS), and thyroid hormones have been considered as the most important players in the development of cardiovascular complications of metabolic disorders [3,4,5,6]. Recently, experimental and clinical studies have provided evidence that vasopressin, which exerts a wide spectrum of regulatory functions, should be included in this group. Vasopressin can act either directly on target cells or indirectly through interactions with several other regulatory systems. Its secretion is effectively altered by glucose, insulin, and other pancreatic hormones [7,8,9]. Moreover, it has been shown that its action is altered in metabolic disorders, such as diabetes mellitus, diabetes resistance, and obesity [10,11,12,13,14,15,16]. With regard to obesity, it is worth noting that vasopressin, and specifically stimulation of V1aR, exerts an anorexigenic action [17,18,19]. Moreover, patients with bulimia nervosa respond with lower blood AVP concentrations during osmotic stimulation [20].

Several studies give attention to altered interactions of vasopressin and insulin in metabolic diseases and inflammatory processes associated with oxidative stress [21,22,23,24]; however, the knowledge in this field is not yet sufficient. The purpose of the present narrative review, based on a PubMed search related to vasopressin, metabolism, and the cardiovascular system, is to summarize current information on the regulation of the secretion and action of arginine vasopressin (AVP) and insulin in context of their interactions in the regulation of blood pressure, tissue metabolism, and oxidative stress and to expose negative consequences of inappropriate cooperation of these two hormones in metabolic diseases, especially in obesity and diabetes mellitus.

## 2. Main Components of Vasopressin System

Arginine vasopressin (AVP) and vasopressin-like peptides are synthesized by mammals and other vertebrates as well as by some invertebrate species [25,26,27,28,29]. The AVP gene encodes sequences of vasopressin, neurophysin II, and copeptin peptides, which are released in equimolar quantities [30,31]. [Arg^8^] Vasopressin (AVP) is a nine-amino acid peptide, synthesized mainly in the magnocellular and parvocellular neurons of the supraoptic (SON) and paraventricular (PVN) nuclei of the hypothalamus. Synthesis of AVP and of its analogs, as well as identification of vasopressin receptors, accelerated rapid research on the physiological and pathophysiological role of this peptide [25,26,29]. In humans, the studies markedly intensified after the introduction of copeptin as a surrogate of AVP. Copeptin is a 39-amino acid peptide, forming the C-terminal portion of the pre-pro-vasopressin molecule, which is secreted together with AVP in equimolar amounts and is more stable than AVP. Frequently, copeptin serves as a convenient marker of vasopressin [32,33,34].

Vasopressin and vasopressin receptors form a highly organized vasopressinergic system, composed of many subsystems located in the brain and peripheral organs. AVP subsystems play an essential role in the regulation of body fluid composition, blood pressure, metabolism, body temperature, reproduction, behavior, learning, and memory. Inappropriate function of AVP subsystems dysregulates function of the whole vasopressin system and results in significant disturbances of homeostasis [8,29,35,36,37].

### 2.1. Synthesis and Release of Vasopressin

In mammals, the main groups of AVP synthesizing cells are located in the hypothalamic nuclei. Vasopressin is synthesized in neurons of the paraventricular nucleus (PVN), the supraoptic nucleus (SON), and the suprachiasmatic nucleus (SCN), and it is subsequently transported by axonal projections to the posterior pituitary, where the projections form abundant intimate contacts with vessels. As discussed in other studies, synthesis and release of AVP are regulated by body fluid osmolality, signals from cardiovascular receptors and chemoreceptors, tissue hypoxia, oxidative stress, hypoglycemia, and several types of stressors engaging rich sets of neurotransmitters and hormones [22,23,29,35,37,38,39,40,41,42]. Some studies indicate that activation of the SCN neurons plays an essential role in the regulation of the inhibitory input to the sympathetic preautonomic neurons in the PVN that are involved in the regulation of hepatic glucose production [43].

Moreover, multiple vasopressinergic neurons send projections to the median eminence, forebrain, midbrain, brain stem, and spinal cord, where they cooperate with other neurons engaged in the control of basic homeostatic functions and participate in the regulation of learning and memory processes, pain, and sensitivity to stress [8,35,44,45,46,47,48,49,50,51]. Presumably, the brain vasopressinergic neurons are not the only source of AVP in the central nervous system, as it has been shown that vasopressin can be transported backwards from the systemic circulation. The blood capillaries in the regions surrounding the brain ventricles and the aqueduct of Sylvius possess fenestrated endothelium, which allows for penetration of AVP to the cerebrospinal fluid [52,53,54,55].

Experimental studies provide evidence for synthesis of vasopressin in the peripheral organs. AVP mRNA has been demonstrated in the heart, and it has been shown that its expression is elevated in the pressure-overloaded heart, in particular in the coronary endothelial cells and vascular smooth muscle cells [56]. Immunoreactive vasopressin and AVP mRNA have been detected in the human and rat pancreatic extracts and tissue [57,58], as well as in the endocrine pancreatic tumors [59,60].

### 2.2. Vasopressin Receptors

Actions of vasopressin are mediated by V1a receptors (V1aR), V1b receptors (V1bR), and V2 receptors (V2R) [61,62]. V1aR and/or V1bR have been detected in several regions of the brain (the cerebral cortex, the hypothalamus, the pons, the medulla, the cerebellum, and the spinal cord) [63,64,65,66,67,68] and in cells of several peripheral organs and tissues, including the heart and vessels, the chemoreceptors of the carotid bodies, the kidney, the lung, the liver, the pancreas, the gastrointestinal tract, and the adipose tissue [50,66,69,70,71,72,73,74,75,76]. In the brain, the highest expressions of V1bR transcript and protein were found in the subfornical organ (SFO), the organum vasculosum laminae terminalis (OVLT), and the median eminence. They were also identified in the amygdala, the hippocampus, the caudate, the putamen, the cerebellum, the cortex, and the olfactory bulb [50]. V2R were found mainly in the kidney; however, V2R mRNA and/or protein were also demonstrated in the brain, the gastrointestinal tract, the lungs, and the heart, which suggests that they may be synthesized in these organs [76,77,78,79,80].

Interaction of AVP with receptors and Gq subtype of G protein causes phosphorylation of GDP and initiates translocation of the receptor into the cytoplasm and subsequently processes of desensitization and resensitization, with engagement of β-arrestin [81,82]. Activation of AVP receptors is regulated by several factors controlling blood pressure and metabolism, including tissue anoxia and inflammatory processes. In this context, the modulating role of deoxycorticosterone acetate (DOCA), glucocorticoids, aldosterone, Ang II, and vasopressin itself has been most intensively investigated [83,84,85,86]. Decrease in V1aR expression in the liver, lung, kidney, and heart, mediated by proinflammatory cytokines (IL-1β, TNF-α and IFNγ), was found in endotoxemia induced by injection of lipopolysaccharide [87]. Downregulation of V1aR by a high concentration of extracellular glucose was also reported by Tahara et al. [88].

## 3. Vasopressin and Oxidative Stress

Gaseous transmitters, in particular nitric oxide (NO), carbon monoxide (CO), and hydrogen sulfide (SH_2_), are considered essential intrinsic regulatory molecules of the brain and peripheral organs, which regulate blood supply and tissue oxygenation [24,89,90,91]. Under normal conditions, their actions improve supply of oxygen; however, this effect can be reversed in the pathological states engaging the oxidative stress. The oxidative stress intensifies the formation of free radicals (O_2_^−^, NO, peroxynitrite OONO^−^, H_2_O_2_), participating in the process of oxidative posttranslational modification. Uncoupling of endothelial nitric oxide synthase by O_2_^−^ and formation of OONO^−^ are especially well recognized in cardiovascular diseases [92]. Growing evidence indicates that the gasotransmitters and free radicals exert significant impact on the function of the vasopressinergic system through regulation of release of AVP and through modulation of its action at the cellular level [89,90,93,94,95,96,97,98].

### 3.1. Vasopressin and Oxidative Stress in the Brain

The magnocellular vasopressinergic neurons of the SON express neuronal NO synthase (NOS) and heme-oxygenase (HO-1), which are involved in the synthesis of NO and CO, respectively [97,98,99,100]. The intracellular NO has been identified within the SON, and it has been shown that it is able to inhibit the firing activity of these neurons [101]. Studies with intracerebroventricular (ICV) administration of an NOS inhibitor (L-NAME) or NO donors (SIN-1, SNAP) provide evidence that the brain NO, acting directly, inhibits the release of AVP and reduces the stimulatory effect of Ang II on the release of AVP, oxytocine, and atrial natriuretic peptide [102].

It has been shown that in the supraoptic nucleus, CO acts as an excitatory molecule. The action of NO and CO involves modulation of the activity of the GABA inhibitory neurons [87,100,103]. It is likely that NO participates in the regulation of AVP synthesis in the PVN and SON, hampering the excitatory effect of Ang II. Namely, it has been shown that NO prevents upregulation of AVP mRNA expression by Ang II in these nuclei [104]. Reactive oxygen species (ROS) are probably involved in modulation of the activity of vasopressinergic neurons in the SON by the noradrenergic afferents [105].

Several factors engaged in the regulation of vasopressin release, such as hyperosmolality, hypoxia, hemorrhage, and inflammation, interfere with the brain nitrergic system. For instance, in the study of Kadowaki et al. (1994), salt loading, which intensified synthesis of AVP in the PVN and SON and its release from the posterior pituitary, also elevated neuronal synthesis of NO in the PVN and SON [106]. Moreover, inhibition of nNOS synthase through intraperitoneal administration of N-omega-nitroarginine intensified the release of AVP from the posterior pituitary. Thus, it is likely that NO may play a role of an inhibitor, buffering release of AVP during sodium loading [106]. Similarly, water deprivation was found to increase synthesis of AVP in the magnocellular cells of the SON and PVN and to activate NO synthase and heme oxygenase in the SON [95,99,107]. It is also possible that NO may play an essential role in local modulation of vascular tone and neurovascular coupling in the SON arterioles. For instance, it has been shown that application of AVP or weak stimulation of vasopressinergic neurons causes vasoconstriction of SON arterioles, which is mediated by V1aR. At the same time, the osmotic stimulation activates vasodilatory neurons of the SON, which are predominantly nitrergic, and this effect may reduce the vasoconstrictory action of AVP [108].

Hemorrhage, which is a potent stimulus of AVP release, was found to cause significant reduction in nitrogen oxides levels (nitrite NO_2_^−^, nitrate NO_3_^−^) in the PVN, amygdala, bed nucleus of the stria terminalis, and periaqueductal gray [109]. It has also been shown that application of N-methyl-D-aspartate (NMDA), which is a glutamate receptors antagonist, significantly magnifies blood AVP concentration, which suggests thereby that NMDA subtype glutamate receptors play a significant role in suppressing the systemic release of AVP during bleeding [109]. The presence of NMDA subunits in nNOS-containing PVN neurons and decrease in their functionality were described in mice undergoing chronic intermittent hypoxia. Chronic intermittent hypoxia also elicited reduced formation of the NR1 subunit of NMDA receptors in nNOS containing neurons of the PVN and resulted in arterial hypertension. The authors suggested that the loss of nitrergic neurons in the PVN may play a role in the development of hypertension [110].

It is likely that NO and CO participate in the regulation of inflammatory release of AVP [111]. Experiments on Wistar rats revealed that polymicrobial sepsis causes enhanced production of NO and stimulation of the NO-cGMP pathway in the SON and PVN [112]. Earlier studies performed on the hypothalamic explants provided evidence that NO inhibits release of AVP by IL-1β, which acts as a proinflammatory cytokine [113].

### 3.2. Vasopressin and Oxidative Stress in Peripheral Organs

Inhibition of NO synthesis by systemic administration of NG-nitro-L-arginine (L-NAME) increases plasma AVP elevation induced by intravenous administration of IL-1β in normotensive Sprague Dawley rats [114]. There is also evidence that NO regulates sensitivity of cells to AVP in the peripheral organs. In this line, it has been shown that in some vascular beds, vasopressin causes vasodilation that is mediated by stimulation of either V1R or V2 receptors, and in both instances, the vasodilatory effect is mediated by NO [47,77,115,116]. Experiments on neonatal cardiac myocytes show that vasopressin, specifically stimulation of V1aR, enhances inducible NOS expression and NO synthesis, which is associated with stimulation of IL-1β and activation of protein kinase C [117]. Experiments on cardiomyocytes of adult Sprague Dawley rats revealed that AVP exerts several actions, including stimulation of collagen synthesis, activation of iNOS, enhanced production of NO, and activation of nuclear factor kappa B (NF-kappa B). Accordingly, it has been suggested that AVP-induced activation of the NF-kappa B–NOS pathway may contribute to development of myocardial fibrosis [118]. It should also be noted that NO counteracts the procoagulatory action of vasopressin in the heart [119].

It is likely that vasoconstrictory/vasodilatory interactions of AVP and NO in peripheral vessels are modulated by TNF-α and other cytokines. For instance, experiments on isolated arterioles of hamsters showed that TNF-α potentiates the vasoconstrictory effect of vasopressin and eliminates its vasodilatory action mediated by NO. It has been suggested that in pathological states involving TNF-α overproduction, there may be a reduction in the vasodilatory potency of vasopressin, which is mediated by NO [120]. It is likely that in human subjects suffering from inflammation induced by administration of lipopolysaccharide (LPS), the oxidative stress and increased production of ROS may account for vasoconstrictory hyporesponsiveness to vasopressin and other vasoconstrictors [91].

Experiments on primary human lung microvascular endothelial cells (HLMVEC) have shown that application of AVP or V2R agonist desmopressin (dDAVP) increases the expression of NOS and NO release in these cells [77]. Vasopressin can also exert a local V2R-mediated vasodilatory effect in the kidney. For instance, stimulation of V2R by application of an agonist has been found to enhance production of NO in the renal medullary interstitium, which presumably mediates the elevation of the renal medullary blood flow [121,122,123]. Other studies performed on rabbits have shown that desmopressin (dDAVP, V2R agonist) causes vasodilation of renal afferent arterioles and reduces the vasoconstrictory potency of norepinephrine. As these effects could be reduced by administration of L-NAME, it has been suggested that the vasodilatory effect of desmopressin was mediated by NO [124]. Studies performed on cultured rat glomeruli cells have shown that stimulation of V1R by vasopressin inhibits LPS-induced and IL 1β-mediated activation of iNOS and increases nitrite production [125]. These findings suggested that regulation of NO synthesis by AVP in the kidney may be significantly altered in the inflammatory states. Studies on human subjects also draw attention to the significant role of NO in the regulation of antidiuretic action of vasopressin. It has been shown that administration of tolvaptan, which is a selective V2R antagonist [126,127], less effectively antagonizes the effect of AVP on renal water and sodium absorption when it is delivered during inhibition of NO synthesis [128]. Furthermore, it has been reported that inhibition of NOS synthesis in the kidney causes upregulation of aquaporin-2 (AQP-2) expression, which suggests that overproduction of NO may play a role in the phenomenon of renal escape from antidiuresis [129].

In the portal-systemic collaterals of hepatic vessels, vasopressin causes vasoconstriction via stimulation of V1aR, and this effect can be significantly potentiated by inhibition of NO synthase [130]. It is likely that overproduction of NO plays a significant role in the hyposensitivity of hepatic vessels to vasopressin, which is observed in portal hypertension. In support of this assumption are findings showing that chronic inhibition of NO production by L-NAME ameliorates responsiveness to AVP in portal hypertensive rats [131]. It is likely that enhanced release of NO is responsible for hyposensitivity to systemic administration of AVP analogue—glypressin, which is observed in patients with portal hypertension and in hepatic cirrhosis [132]. Experiments on cultures of isolated hepatocytes showed that increased iNOS expression decreases vasopressin responsiveness and reduces V1aR level in these cells [133].

## 4. Vasopressin and Regulation of Metabolism

**Figure 1:**
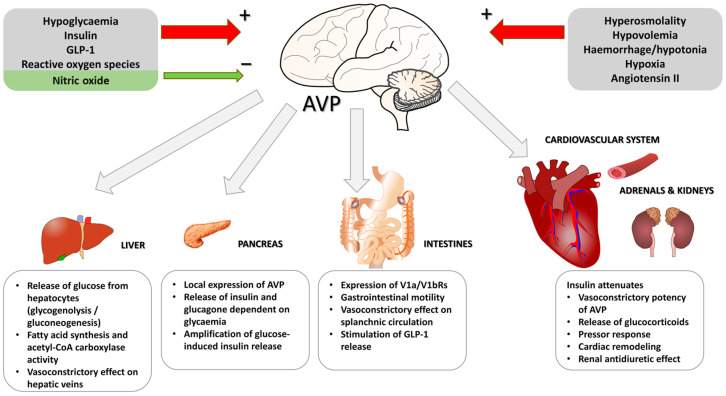
Role of vasopressin in regulation of metabolism and insulin secretion. AVP—arginine vasopressin, GLP-1—glucagon-like peptide-1. See text for other explanations.

Early studies addressing the role of vasopressin in regulation of metabolic processes revealed that AVP elevates release of glucose from hepatocytes and that this effect results from enhanced glycogenolysis and gluconeogenesis [134,135,136,137]. Subsequently, it has been shown that AVP also exerts indirect metabolic effects executed in cooperation with insulin, glucagon, and other glucostatic and lipostatic hormones [7,33,138,139] (Figure 1).

### 4.1. Metabolic Effects of Vasopressin

Experiments on isolated hepatocytes revealed that acting on V1R AVP stimulates phosphorylase, increases inositol triphosphate (InsP3), and enhances mobilization of calcium from intracellular stores. Altogether, direct actions of vasopressin in the liver promote glycogenolysis and reduce synthesis of glycogen [140,141,142]. In addition, acting on isolated hepatocytes, AVP stimulates fatty acid synthesis and acetyl-CoA carboxylase activity, thereby modulating lipogenesis [143]. Subsequently, it has been shown that the hepatic glycogen breakdown, inhibition of fatty acid synthesis, and cholesterol synthesis are less intensively affected by AVP in genetically obese (*ob*/*ob*) mice than in control mice [134,135,144]. The direct metabolic effects of vasopressin in hepatocytes were also attenuated in genetically obese (*fa*/*fa*) Zucker rats [145].

Some evidence indicates that vasopressin may influence metabolism of adipocytes and myocytes. For instance, it has been shown that incubation of isolated rat adipocytes with AVP significantly increases the activity of membrane-bound protein kinase C (PKC) [146]. Immunoreactive AVP has been detected in human fetal and neonatal skeletal muscle [147]. Moreover, it has been shown that I.6 myogenic cells culture express V1aR and V1aR, which are significantly upregulated during the process of post-injury muscle regeneration. Thus, it is likely that AVP plays a positive role in muscle regeneration [148].

There is also evidence that the regulation of metabolism by centrally acting vasopressin may be altered in obesity. Hence, it has been shown that infusion of AVP to the third cerebral ventricle increases the sympathetic firing rate in brown adipose tissue of lean and obese rats, and that the response is significantly greater in the obese individuals [149].

**Figure 2:**
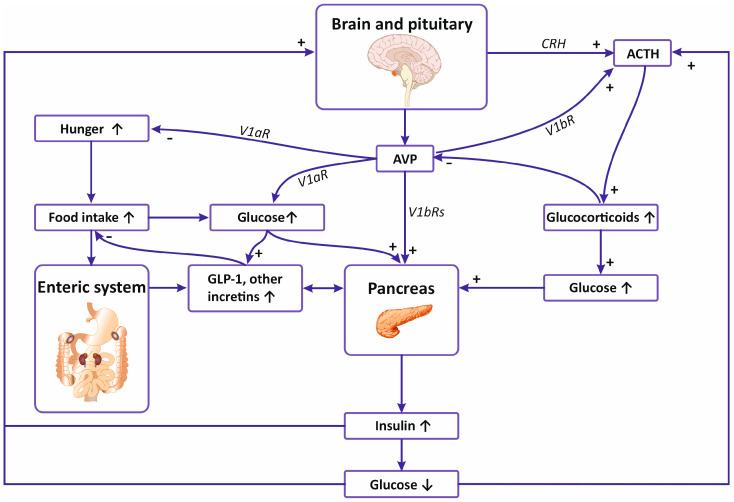
Interaction of vasopressin and insulin in the brain, pancreas, and enteric system. ACTH—adrenocorticotropic hormone, AVP—arginine vasopressin, CRH—corticotropin-releasing hormone, GLP-1—glucagon-like peptide-1. See text for other explanations.

Abundant evidence indicates that AVP contributes significantly to the regulation of metabolism, controlling the release and action of the key pancreatic hormones (Figure 2). Vasopressin mRNA has been identified in the pancreas, and AVP was able to stimulate release of glucagon and insulin from the isolated pancreatic islets and from the in situ perfused pancreas [7,150,151,152]. Recently, it has been shown that AVP enhances release of insulin by glucose in the pancreas via potentiation of paracrine production of glucagon, which subsequently activates GLP-1 receptors [153]. Experiments on mouse islets showed that AVP significantly amplifies glucose-induced insulin release [12]. Furthermore, it has been shown that it potentiates the effect of ACTH on release of insulin from β pancreatic cells [154]. Parallel experiments on mice revealed that the mice deprived of V1bR responded with significantly lower increases of ACTH and corticosterone during acute immune stress induced by administration of bacterial LPS or by intoxication with ethanol, which suggested that stimulation of V1bR is essential for appropriate regulation of the hypothalamic-pituitary-adrenal axis during inflammatory stress [155]. Studies analyzing expression of AVP receptors in the human pancreas demonstrated the presence of V1bR mRNA and V1bR binding sites in β-insulin, α-glucagon, and somatostatin cells [73]. It is likely that the process of release of insulin by vasopressin is associated with depolarization of β-cells, as experiments on the insulin-secreting cell line (RINm5F) revealed that AVP closes ATP-sensitive potassium channels (K_ATP_) and induces depolarization, which is followed by an increase in intracellular calcium concentration [Ca^2+^]_i_ [156]. Moreover, it has been shown that the effect of vasopressin on the release of pancreatic hormones involves elevation of calcium influx [73]. Stimulation of insulin release by vasopressin was confirmed in the experiments on clonal hamster beta cells, showing that AVP releases insulin directly from beta cells through action mediated by InsP3, and its action can be inhibited by somatostatin [157]. Studies on hamster In-R1-G9 cells of glucagonoma showed that AVP increases release of glucagon, and its effect is mediated by V1bR [158].

Recently, experiments on Sprague Dawley rats and lean Zucker rats confirmed that acute AVP administration causes hyperglycemia, mediated by V1aR receptors, and that this is followed by secretion of insulin and a decrease in blood glucose level. However, they also showed that chronic AVP infusion may produce hyperglycemia, which is not associated with hyperinsulinemia. These findings suggested that some adaptive processes occur in the interactions of AVP with insulin during chronic exposure to vasopressin [159]. It is also likely that, acting in the pancreatic cells, AVP may play the role of a double modulator of glucose homeostasis, whose action is adjusted to extracellular glucose concentration, as it has been shown that at hypoglycemic concentrations, AVP enhances release of glucagon, whereas at hyperglycemic levels, it promotes release of insulin [7].

The importance of vasopressin receptors for regulation of glucose tolerance and glucagon secretion has been investigated in genetically linked models. Expression of AVP mRNA was found to be elevated in the brains of *db*/*db* mice, which is a genetic model of type II diabetes [160]. Subsequently, experiments using the hyperinsulinemic-euglycemic clamp model revealed that V1aR-deficient rats are less sensitive to insulin, whereas V1bR-deficient mice maintained on a high-fat diet manifest impaired glucose tolerance and develop overt obesity [71,161,162]. It has been shown that plasma glucose, insulin, and glucagon levels are lower in *V1bR*^−/−^ mice than in wild-type mice. In addition, V1bR-deficient mice manifested lower plasma glucose and insulin levels during glucose-tolerance tests, which indicated greater sensitivity to insulin. The authors also provided evidence for elevated phosphorylation of insulin-dependent Akt in *V1bR*^−/−^ mice and suggested that adipocytes may be an essential site of insulin and vasopressin interactions [161]. The importance of stimulation of both types of V1 AVP receptors for morphology of β-cells and transdifferentiation of pancreatic β-cells to α-cells was evaluated in two models of streptozotocin (STZ) diabetic transgenic mice (*Ins1Cre*^−/+^; *Rosa26-eYFP* and *GluCreERT2 Rosa26-eYFP*) that were chronically treated with vasopressin analogue (Ac3IV; Ac-CYIQNCPRG-NH2), activating both V1aR and V1abR. The authors confirmed that STZ diabetic transgenic mice manifest enhanced transdifferentiation of pancreatic β-cells to α-cells and showed that this effect could be partly reversed by administration of Ac3IV [163]. A significant role of V1R in the regulation of β-cells was confirmed in *Ins1Cre*^−/+^; *Rosa26-eYFP* mice chronically treated with hydrocortisone. The experiments showed that administration of Ac3IV prevents hydrocortisone-induced transdifferentiation of pancreatic cells and promotes a shift of the islet number towards control levels [164]. In addition, enhanced glucose tolerance has been described in Brattleboro rats [165]. Other studies revealed that vasopressin elevates expression of 11-β-hydroxysteroid-dehydrogenase-type 2 (11-β-HSD2) and may therefore play a role in the development of insulin resistance [11].

### 4.2. Interactions of Vasopressin with Insulin and GLP-1

Some evidence indicates that vasopressin is a physiological substrate for insulin-regulated aminopeptidase (IRAP) and that insulin may play a role in the clearance and turnover of vasopressin. In particular, it has been shown that in the IRAP-deficient mice (*IRAP*^−/−^), cleavage of vasopressin by adipocytes and skeletal muscle is markedly reduced, which results in significant elevation of plasma AVP level [166]. Expression of IRAP in the skeletal muscle cells and adipocytes was found to be upregulated by insulin [167]. It has been suggested that insulin resistance and high insulin levels increase IRAP activity and alter AVP turnover, which leads to increased synthesis of vasopressin, and may play a role in the development of metabolic syndrome [168]. Recently, a high fat diet was shown to decrease expression of V1aRs and to increase V1bRs in the retroperitoneal adipose tissue in rats [169]. Significantly elevated expression of AVP mRNA was found in the brains of *db*/*db* mice [160]. Other studies revealed that vasopressin elevates expression of 11-β-hydroxysteroid-dehydrogenase-type 2 (11-β-HSD2) [170] and may therefore play a role in the development of insulin resistance [11].

It is known that AVP exerts a significant vasoconstrictory effect on hepatic, pancreatic, and gastrointestinal vessels via V1aR and that its vasoconstrictory potency is altered under pathological conditions, especially those associated with inflammation [171,172,173,174,175]. Vasopressin also regulates gastrointestinal motility, which may play a role in absorption of nutrients. Acting locally in the gastrointestinal tract, AVP increases contractions of the smooth muscles of the gastrointestinal wall by binding to its V1aRs [176,177]. Acting on the central V1bRs, it alters intestinal peristalsis, and this action appears to be essential in chronic stress [178,179]. The gastric emptying and motility of the gastrointestinal tract determines the load of nutrients reaching the intestine and may thereby affect the gut-pancreas axis activity, release of insulin, and post-prandial glucose levels [180,181].

Recent evidence points to the interactions of vasopressin with glucagon-like peptide type 1 (GLP-1) and other incretin hormones [153]. GLP-1 is a key incretin hormone released from the gastrointestinal tract in response to glucose and peptides present in the chyme [182]. It potentiates insulin release from the pancreatic β-cells in the presence of glucose—the phenomenon termed “incretin effect”, which plays an important role in prevention of excessive elevation of plasma glucose levels [182,183]. In addition, GLP-1 acting on its receptors expressed in the carotid body attenuates sympathetic activity induced by high plasma glucose levels [184]. Accumulating evidence indicates that GLP-1 is also the main food intake inhibiting incretin hormone of the gut-brain axis [185]. The physiological effects of incretin hormones initiated studies on a new class of plasma glucose-lowering and anti-obesity drugs, which have been successfully tested in clinical trials [186].

It has been shown that acting on V1bR vasopressin stimulates release of GLP-1 from the human and murine L-cells of the colon [187,188]. Interestingly, the L-cells manifested inverse sensitivity to glucose and AVP, i.e., the L-cells that were most sensitive to vasopressin were least sensitive to glucose [188]. Previous studies have shown that GLP-1 stimulates synthesis of AVP mRNA in the hypothalamic neurons and enhances its release into the bloodstream [189,190]. Recently, it has been shown that GLP-1 receptor agonists enhance antidiuretic effects of vasopressin analog desmopressin [191]. Together, these observations indicate that there is a reciprocal positive feedback interaction between vasopressin and GLP-1 secretion, with one hormone potentiating release/actions of the other. However, given the opposing effects of GLP-1 and vasopressin on plasma glucose, it is not yet known if vasopressin-GLP-1 interactions play a significant role in the regulation of insulin release and in the regulation of glucose homeostasis.

Finally, it should be noted that AVP may participate in the regulation of glucose homeostasis through other not yet fully recognized mechanisms. For instance, it has been shown that rat administration of AVP into the NTS elevates blood glucose level and increases brain arterio-venous glucose difference; these effects could be prevented by blockade of V1R [192].

## 5. Role of Glucose and Insulin in Regulation of Vasopressin Secretion

It has long been thought that insulin does not play a significant role in the regulation of the brain neuronal processes because it does not penetrate the blood–brain barrier (BBB). Currently, it has been established that insulin can be transported to the brain by a saturable transport [193,194,195]. There is also evidence for neuronal and glial synthesis of insulin and insulin receptor [196,197,198].

In 1977, Baylis and Heath reported that systemic administration of insulin in hypoglycemic doses causes in human subjects significant elevation of plasma AVP level [199]. The stimulatory effect of insulin on AVP secretion was confirmed in several other species [10,138,200,201] and in patients with type 1 diabetes mellitus [202]. It has been suggested that insulin modulates vasopressin secretion and that this effect is exerted mainly during the cephalic phase of insulin release [203].

The presence of insulin receptors in the brain regions innervated by vasopressinergic neurons suggests the possibility of direct interactions of insulin and vasopressin in the brain [204,205]. Studies on Zucker diabetic fatty rats, which are used as an experimental model of human type 2 diabetes, showed that these rats manifest significantly higher expression of AVP in the PVN and SON than the control rats [206]. Direct stimulatory effect of insulin on AVP release was confirmed by the study of Song et al. (2014), who found that application of insulin together with glucose into the rat hypothalamo-neurohypophyseal explants containing the SON causes significant increase in AVP from the SON neurons [207]. In parallel, it has been shown that disruption of InsR signaling in the hypothalamus of *InsR* knockout mice (IRNKx2.1) results in significant changes in mRNA expression of AVP in the hypothalamus under baseline conditions as well as in decreased expression of AVP peptide in the pituitary during restrain stress [208]. Recently, the study of Paiva and Leng (2020) revealed that intravenous administration of insulin effectively stimulates vasopressin neurons in the SON, which indicates that systemically released insulin may also have an influence on the release of AVP from the hypothalamic neurons [209].

Interestingly, obese Zucker rats chronically exposed to intraperitoneal infusion of vasopressin manifested significantly higher fasting insulinemia and higher insulin resistance index than their lean counterparts and control laboratory rats. In addition, prolonged administration of vasopressin elicited significantly higher expression of hepatic V1aR mRNA in the obese rats. In the same study, lean rats responded with higher fasting glycemia and fourfold higher insulin resistance index to chronic AVP administration in comparison to the rats not treated with AVP. Moreover, blockade of V1aR partly reduced glucose intolerance in Zucker obese rats [210]. It should also be noted that experiments on rats with streptozotocin-induced diabetes mellitus associated with hyperglycemia and hyperosmolality disclosed elevated AVP secretion and reduced V1aR mRNA expression in the liver [211].

Studies on direct engagement of insulin in the regulation of the brain vasopressin system are complicated by interference of the post-insulin hypoglycemia. In addition, it has been shown that enhanced secretion of vasopressin during insulin-induced hypoglycemia is associated with elevated release of ACTH [200,201,208]. Accordingly, it has been suggested that parallel stimulation of AVP and ACTH secretion synergistically counteract post-insulin hypoglycemia [202]. Recently, it has been suggested that insulin, glucagon, and vasopressin also interact directly in the pancreas at the cellular level. Studies on mice revealed the presence of V1bR mRNA at the pancreatic α-cells and showed that enhanced release of AVP and stimulation of V1bR play a significant role in the elevation of glucagon release during insulin-mediated hypoglycemia [139].

In humans, a significant impact of insulin-induced hypoglycemia on AVP secretion has been documented in multiple studies using estimations of plasma copeptin level, as a surrogate marker of vasopressin secretion [11,13,139,212]. It has been shown that in patients with diabetes mellitus, intravenous administration of insulin causes elevation of copeptin in the blood that is associated with symptoms of hypoglycemia [11,213].

Experimental studies have provided evidence that type 1 diabetes mellitus suppresses the stimulatory effect of vasopressin on the release of glucagon during hypoglycemia [139]. There is also evidence that altered relations of copeptin with insulin may be an indicator of developing insulin resistance [212]. In addition, in patients with newly detected diabetes, a negative correlation was found between plasma copeptin level and stress coping ability that was evaluated by the sense of coherence scale [13].

Pathological states occurring with insulin resistance are associated with oxidative stress [214,215], which may affect interactions between vasopressin and insulin. Hyperglycemia was found to intensify oxidative stress in endothelial cells, and these effects could be partly reversed by administration of insulin [216,217]. Experiments on Sprague-Dawley rats with streptozotocin-induced diabetes showed that the diabetes results in enhanced oxidation of lipid, glucose, and protein that is associated with reduced expression of eNOS, nNOS, and nitrotyrosine and can be effectively reduced by administration of insulin [218]. It has also been shown that inhibition of NO synthesis enhances stimulation of glucose-induced insulin secretion, whereas applications of NO donor (SNAP) or proinflammatory cytokines such as TNF-α, IL-1β, and IFNγ decrease the viability of insulin-secreting cells (INS-1) and reduce the excitatory effects of cholinergic stimulation on glucose-induced insulin secretion [219,220]. Immunofluorescence studies on pancreatic β cells of the rat provided evidence that NO and CO may play an opposite role in the regulation of basal secretion of insulin, with NO acting as a stimulatory factor and CO exerting an inhibitory effect [221]. It has also been shown that SH_2_ inhibits glucose-induced release of insulin in pancreatic β-cells and protects the pancreatic islets from apoptotic cell death, which is induced by high glucose concentration [222].

It is likely that insulin significantly affects vasoconstrictory potency of vasopressin. Experiments on cultured vascular smooth muscle cells have demonstrated that insulin in concentrations of 1 and 100 mU/mL shifts to the right dose-response curves depicting AVP-dependent rises in [Ca^2+^]_i_, and decreases Ca^2+^ influx [223]. As vasopressin plays a significant role in the regulation of blood flow through the liver, gastro-intestinal tract, and adipose tissue [171,172,173,174,175], it is possible that changes in the vasoconstrictory potency of AVP by insulin may have an impact on blood flow in the gastrointestinal tract and in consequence on the supply of substrates in this region.

## 6. Clinical Aspects of Vasopressin-Insulin Interactions in Metabolic Disorders

Substantial evidence indicates that insulin-vasopressin interactions may be significantly altered in obesity and diabetes mellitus. Responses of vasopressin to insulin-induced hypoglycemia were found to be significantly lower in obese men than in normal weight men [224,225]. It was also shown that although in patients with diabetes mellitus, the insulin-dependent hypoglycemia elevated blood vasopressin concentration [226], the release of AVP was markedly smaller in diabetic patients with asymptomatic insulin-induced hypoglycemia than in the healthy subjects [227]. The post-mortem immunohistochemical examination of human hypothalamic tissue revealed significantly lower numbers of AVP immunoreactive neurons and astroglial cells in the SCN of subjects with type 2 diabetes than in the SCN of the control individuals [228]. Nonetheless, several small studies showed that plasma vasopressin levels are significantly increased in patients with insulin-treated diabetes mellitus and hyperglycemia [229] or in patients with uncontrolled diabetes associated with marked hyperglycemia, plasma hyperosmolality, and polyuria [16,230,231,232]. High plasma vasopressin levels were primarily dependent on enhanced vasopressin secretion at given serum sodium concentrations rather than on plasma hyperosmolality [229,231]. In fact, higher osmolality values were needed for secretion of AVP and urine concentration in response to dehydration in diabetic patients with poorly controlled hyperglycemia than in patients with well-controlled diabetes or healthy subjects [233]. Recently, it has been shown that administration of gliflozins—sodium-glucose transporter type 2 (SGLT2) inhibitors—to patients with type 2 diabetes mellitus induces persistent osmotic diuresis, which in turn promotes the release of AVP (evaluated by plasma copeptin levels), which by its antidiuretic activity counteracts osmotic diuresis-induced dehydration [234]. Despite the above findings pointing to increased plasma vasopressin levels and altered secretion of the hormone in response to natriuretic/osmotic stimuli, vasopressin release triggered by hemodynamic factors, especially orthostatic hypotension, was found to be attenuated in patients with diabetes mellitus type 2, possibly due to dysfunctional afferent signaling from arterial baroreceptors [235,236].

Clinical studies performed on North European, African-American, and non-Hispanic White American cohorts revealed significant association of elevated blood copeptin levels, which is a robust marker of vasopressin secretion, with insulin resistance and with the risk of development of diabetes [6,38,40,237,238]. Another multicenter study showed that after adjustment for age and sex, plasma copeptin levels are positively correlated with body mass index, fasting plasma glucose, and insulin and triglycerides levels, whereas they are inversely correlated with high-density lipoprotein cholesterol. The data suggested a significant causal link between the copeptin level and a set of symptoms characteristic of the pathophysiology of metabolic syndrome and insulin resistance [238]. Similarly, data from the Swedish population revealed that copeptin level can be used as a predictor of development of diabetes, independently of other loading factors, including fasting glucose and insulin and family history of diabetes mellitus [40,239]. There is evidence that associations between copeptin level and symptoms of insulin resistance may differ in different populations and that they are sex-dependent, with the connection being more significant in men than in women [240,241,242].

In patients with type 1 and type 2 diabetes mellitus, there are also associations of plasma vasopressin and its surrogate marker copeptin levels with the development of chronic kidney disease. Namely, it was found that plasma copeptin level in these patients is positively associated with urinary albumin excretion and albuminuria [243,244,245,246] and decline in renal function [247,248,249]. Moreover, in large cohorts of patients with type 2 diabetes, a higher plasma concentration of copeptin was positively associated with major cardiovascular events independently of renal parameters [249].

It is highly probable that specific genotypes of vasopressin-related genes may predispose to the development of metabolic disorders. The prevalence of hyperglycemia and type 2 diabetes in humans with certain AVP-neurophysin II genotypes has been shown in a long-term follow-up study on a large cohort of the French population (5215 participants) [241]. The study revealed significant associations of the CC genotype of rs6084264, the TT genotype of rs2282018, the C-allele of rs277038, and the CC genotype of rs1410713 with an increased risk for fasting hyperglycemia and type 2 diabetes mellitus. Noteworthy, the associations were significant in male but not in female participants [241]. Further studies are necessary to elucidate if targeting vasopressinergic system may effectively diminish the cardiometabolic disorders in human patients.

In summary, frequent coincidence of cardiovascular and metabolic diseases suggests similar molecular backgrounds of these disorders. Several studies indicate that vasopressin cooperates with insulin in several planes and may play the role of a link in the development of hypertension and diabetes mellitus. In this review, we summarize evidence that direct and indirect metabolic effects of vasopressin engage both V1a and V1b receptors and that AVP exerts its actions in the brain, cardiovascular system, liver, adipose tissue, autonomic nervous system, kidney, and pancreas (Figure 1 and Figure 2). The interactions between AVP and insulin are significantly affected in hypertension, cardiac failure, obesity, insulin deficiency, and insulin resistance.

## 7. Conclusions

The survey of literature allows the formulation of the following conclusions: 1. AVP regulates metabolism through direct actions exerted at the cellular level in target tissues and through interactions with insulin, GLP-1, catecholamines, and glucocorticoids. 2. Under normal conditions, AVP exerts direct metabolic actions in the liver, causing hyperglycemia; however, it also increases release of insulin, which counteracts the hyperglycemic effect and helps restore plasma glucose level to the normal values. 3. Regulation of metabolic processes by AVP is significantly modulated by gasotransmitters and free radicals. 4. Secretion of vasopressin is stimulated by insulin and by hypoglycemia. 5. The stimulatory effect of insulin on AVP secretion and the potency of insulin to reduce AVP-induced hyperglycemia are diminished in diabetes mellitus and insulin resistance, which may result from inappropriate generation of gasotransmitters and free radicals. 6. Clinical studies indicate that elevated concentrations of plasma AVP, or its surrogate marker copeptin, are associated with a higher risk of diabetes mellitus and with aggravation of renal and cardiovascular complications of this disease.

## Data Availability

Not applicable.

## Abbreviations

AVP = arginine vasopressin, ACE = angiotensin-converting enzyme, ACTH = adrenocorticotropic hormone, Ang = angiotensin, AQP-2 = aquaporin of type 2, [Ca^2+^]_i_—intracellular calcium concentration, cGMP = cyclic guanosine monophosphate, CO = carbon monoxide, CoA = coenzyme A, CRH—corticotropin-releasing hormone, dDAVP = desmopressin, DOCA = deoxycorticosterone acetate, GDP = guanosine diphosphate, GLP-1 = glucagon-like peptide type 1, ICV = intracerebroventricular, HO-1 heme oxygenase, H_2_O_2_ = hydrogen peroxide, HLMVEC = human lung microvascular endothelial cells, 11-β-HSD2 = 11-β hydroxysteroid dehydrogenase of type 2, IFNγ = interferon of type γ, IL-1β = interleukin of type 1β, INS-1 = insulin-secreting cells of type 1, INsP3 = inositol triphosphate, IRAP = insulin-regulated aminopeptidase, K_ATP_ = ATP-sensitive potassium channels, L-NAME = NG-nitro-L-arginine methyl ester, LPS = lipopolysaccharide, NF-kappaB = nuclear factor of type kappa B, NMDA = N-methyl-D-aspartate, NO = nitric oxide, NOS = nitric oxide synthase, OONO^−^ = peroxynitrite, OVLT = organum vasculosum laminae terminalis, PKC = protein kinase C, PVN = paraventricular nucleus, RAS = renin-angiotensin system, ROS = reactive oxygen species, SCN = suprachiasmatic nucleus, SFO = subfornical organ, SGLT2—sodium-glucose transporter type 2, SH_2_ = hydrogen sulfide, SON = supraoptic nucleus, TNF-α = tumor necrosis factor of type α, V1aR = vasopressin receptor of type 1a, V1bR = vasopressin receptor of type 1b, V2 = vasopressin receptor of type 2.
